# The epidemiology of polymyalgia rheumatica in primary care: a research protocol

**DOI:** 10.1186/1471-2474-13-102

**Published:** 2012-06-15

**Authors:** Sara Muller, Samantha Hider, Toby Helliwell, Joanne Bailey, Kevin Barraclough, Louise Cope, Bhaskar Dasgupta, Rebecca Foskett, Rhian Hughes, Zoe Mayson, Charlotte Purcell, Edward Roddy, Simon Wathall, Irena Zwierska, Christian D Mallen

**Affiliations:** 1 Arthritis Research UK Primary Care Centre, Primary Care Sciences, Keele University, Keele, Staffordshire, ST5 5BG, UK; 2 Painswick Surgery, Hoyland House, Painswick, Gloucestershire, GL6 6RD, UK; 3 Southend University Hospital, Southend University Hospital NHS Foundation Trust, Prittlewell Chase, Westcliff-on-Sea, Essex, SS0 ORY, UK; 4 Primary Care Research West Midlands North, Unit 2, Badhan Court, Telford, TF1 5QX, UK

**Keywords:** Polymyalgia rheumatica, Primary health care, Cohort studies, Health surveys, Medical records

## Abstract

**Background:**

Polymyalgia Rheumatica (PMR) is the commonest inflammatory condition seen in older patients in primary care. To date, however, research has been focused on secondary care cohorts rather than primary care where many patients are exclusively managed. This two year prospective inception cohort study of PMR patients will enable us to understand the full spectrum of this condition.

**Methods:**

Patients diagnosed with PMR in primary care will be identified via Read codes and mailed a series of postal questionnaires over a two-year period to assess their levels of pain, stiffness and functioning, as well as medication usage and other health-related and socio-demographic characteristics. In addition, participants will be asked for permission to link their survey data to their general practice electronic medical record and to national mortality and cancer registers.

**Discussion:**

This will be the first large-scale, prospective, observational cohort of PMR patients in primary care. The combination of survey data with medical records and national registers will allow for a full investigation of the natural history and prognosis of this condition in the primary care setting, in which the majority of patients are treated, but where little research on the treatment and outcome of consultation has been undertaken. This will provide information that may lead to improved primary care management of PMR.

## Background

In the UK, Polymyalgia Rheumatica (PMR) is the most common inflammatory rheumatic disease in adults aged 50 years and over, and is characterised by pain and stiffness in the shoulder and hip girdles and an elevated acute phase response [1]. On an international scale, the condition is known to increase with more Northerly latitude [2], but within the UK, PMR has been found to be more common in the South than in the North [3].

Whilst less common than other inflammatory arthropathies, such as Rheumatoid Arthritis, PMR is the commonest inflammatory disorder of older adults. In the UK, the estimated annual incidence is variable, ranging from 0.01 % to 0.08 % in those aged 40 years and over [3-5]. Crowson et al [6] estimate the life-time risk of PMR in the US to be 2.4 % for females and 1.7 % for males. In the same population, the annual incidence has been estimated to be between 0.5 % and 0.6 % [7,8]. With the ageing population [9], the number of patients with PMR is set to rise.

Health care systems vary between countries, but in the UK the majority of patients with PMR are managed exclusively in primary care [5,10]. Despite this, the majority of PMR research has been in the secondary care setting, which is likely to represent those with more severe disease than those managed solely in primary care. GPs are currently unable to offer their patients evidenced-based information regarding the prognosis of their condition, because there has not been a study of the course of PMR in primary care. Indeed, there is so little research examining the long-term prognosis of PMR, that there are no agreed patient-reported outcome measures and only recently has a definition of remission been suggested [11].

The recommended treatment for PMR is low dose corticosteroids, where the dose is tapered over a period of up to two years. This makes PMR one of the most common reasons for the long-term use of steroid therapy [1], and consequently has the potential to put PMR patients at increased risk of steroid-related complications, such as fractures and gastro-intestinal bleeding. Furthermore, there are some data to suggest that patients with PMR have increased mortality rates [12], although other studies have shown no such association [13].

Given the non-specific nature of some of the presenting symptoms such as joint pain and morning stiffness, recent diagnostic guidelines [1] have emphasised the need to exclude alternative diagnoses such as Rheumatoid Arthritis or malignancy, which may initially present in a similar manner and may also improve (at least in the early stages) with corticosteroid treatment. Little is known about the patterns of consultation of such patients or clinical predictors of PMR.

The long-term outcomes of patients with PMR are, largely unknown, particularly in the primary care population. This paper outlines the proposed protocol for an inception cohort of primary care patients receiving a diagnosis of PMR in the UK.

## Objectives

The overall aim of this study is to assess the epidemiology of PMR in general practice.

Specific objectives in order to meet this aim include:

1. Describe the natural history and prognosis of PMR in primary care;

2. Identify the frequency of alternative diagnoses in those originally diagnosed with PMR;

3. Identify appropriate outcome measures for PMR in primary care;

4. Assess the association between PMR disease activity and quality of life;

5. Identify the patterns of PMR-related disability over a two-year period;

6. Describe the use of corticosteroids by patients with PMR over a two year period, and how this relates to pain and stiffness outcomes;

7. Assess clinical practice versus audit standards specified in British Society for Rheumatology [1] and the Royal College of Physicians [14] guidelines.

## Methods/design

This study has received ethical approval from the Staffordshire Local Research Ethics Committee (REC reference number: 12/WM/0021).

### Design

Prospective observational inception cohort recruited in UK primary care.

### Sampling frame

All adults (aged ≥18 years) registered with approximately 200 general practices, presenting at the practice and receiving a new Read-coded diagnosis of PMR between June 2012 and June 2014.

### Patient eligibility

#### Inclusion criteria

· Aged 18 years and over.

· Registered with a participating general practice during the study period.

· First Read-coded consultation for PMR in the last three years occurred during the study period.

· Provided written informed consent to primary care medical record review.

#### Exclusion criteria

· Less than 18 years of age.

· Vulnerable groups, e.g. significant cognitive impairment, dementia, severe/terminal illness.

· Previous PMR diagnostic code in the last three years.

### Recruitment procedure

#### Patient identification

Eligible patients will be identified via one of two methods: Method A and Method B. The different methods will be applied in different practices, according to practice preference and local recruitment systems. In both methods, when a patient consults with a new diagnosis of PMR and the general practitioner (GP) enters an appropriate PMR Read code (N20..), a pop-up window will appear, reminding the GP of the study. This window will request that, if they have not already done so, the GP orders the blood tests recommended for the diagnosis of PMR [1] and that they give the patient a postcard containing details of the study.

In Method A, in addition to the blood test and postcard reminder, the pop-up window will ask the GP to discuss the study with the patient and gain their consent to be contacted by the Research Centre. The GP will then be asked to complete a fax form and return to the Research Centre with the patient’s name, gender, date of birth, address, NHS number, practice identifier and confirmation that he/she has a new diagnosis of PMR. On receipt of the fax by the Research Centre, the patient’s details will be entered into a secure mailing database. The fax form will be stored in a secure filing cabinet.

In Method B, practice staff or staff from the Primary Care Research Network (PCRN) will conduct fortnightly electronic searches of the primary care records in participating practices in order to identify patients with a new diagnosis of PMR.

The patient identification process will not interfere with routine primary care management except in that all participating general practices will be provided with copies of the British Society for Rheumatology guidelines for the management of PMR [1] and encouraged to request the recommended blood tests.

#### Initiating patient contact

Eligible patients identified by Method A will be sent a Study Pack from the Research Centre, containing a cover letter and Participant Information Sheet inviting them to take part and giving further details about the study. The name, contact telephone number and email address of the Principal Investigator will be provided should potential participants have any questions regarding the study. The Study Pack will also contain a Baseline Questionnaire with Consent Form and a stamped addressed envelope. For patients identified through Method B, the study pack will be sent by the PCRN on behalf of the general practitioner and will contain an invitation letter from the practice, rather than the research team.

Participants returning their questionnaire and consent form will be logged on the database. Returning a completed baseline questionnaire will be taken to indicate consent to be mailed the follow-up questionnaires. Consent forms will be stored securely with the fax forms received from GPs.

#### Non-responders

Non-responders to the mailed Study Pack will be sent a Reminder Letter with repeat Baseline Questionnaire after three weeks. Non-responders after this reminder will be assumed not to have consented to take part and will not be contacted again.

### Follow-up procedure

#### Responders to Study Pack not consenting to medical record review

Participants who return the completed baseline questionnaire but do not give consent to medical record review will receive mailings of further questionnaires as described below, but will not have their medical record accessed.

#### Consenting responders

Participants who return their Baseline Questionnaire and provide written informed consent to medical record review will be sent a Follow-up Questionnaires at one, four, eight, 12, 18 and 24 months after Baseline. Their medical records will be accessed as described below.

**Figure 1:**
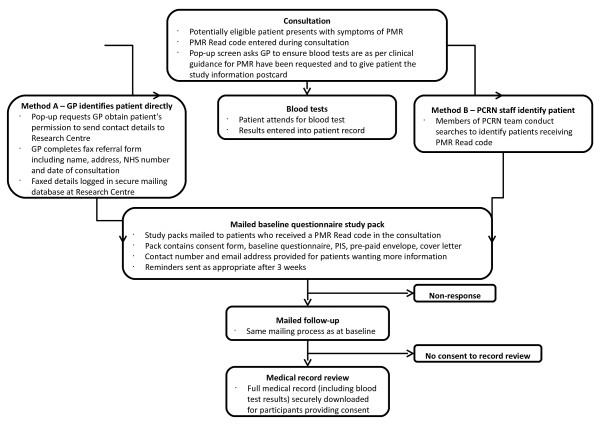
**Flow chart of the recruitment process.** PMR – Polymyalgia Rheumatica**.** GP – General Practitioner. PCRN – Primary Care Research Network. NHS – National Health Service. ONS – Office for National Statistics. PIS – Patient Information Sheet. EMR – Electronic Medical Record.

A flowchart of the process of recruitment and follow-up is provided in Figure 1.

### Data collection

#### Medical record review

Where electronic data quality and practicalities allow, full electronic medical records of consenting participants will be accessed and securely downloaded to obtain information on symptoms, diagnoses, prescriptions and referrals. This data will be downloaded for the two years prior to diagnosis and for the two years of follow-up after diagnosis. Where electronic coding is not routine practice, or where downloading of data is impractical, a research nurse from the PCRN will visit practices and record salient data using a standardised proforma.

For validation purposes, records will be reviewed by a rheumatologist for the six months following PMR diagnosis. This review will use recorded symptoms, blood test results and examine for alternative diagnoses (e.g. malignancy). This review will be based on recent guidance from European League Against Rheumatism-American College of Rheumatology concerning classification criteria for PMR [15].

#### Self-complete postal questionnaires

The self-complete postal questionnaires will provide information on the descriptive characteristics of participants, outcome measurement, health care utilisation and more detailed prognostic information. The conceptual domains, operational definitions, and empirical measures used are provided in Table 1.

**Table 1:** Self-complete postal questionnaire. Conceptual domains, operational definitions and empirical measures

**Conceptual domain**	**Operational definition**	**Empirical measure**	**Number of items**	Time point
**PMR symptoms**				
PMR-related pain	Level of pain attributed to PMR at time of questionnaire	Numerical rating scale (0–10) of pain intensity	1	ALL
PMR-related stiffness	Level of stiffness attributed to PMR at time of questionnaire	Numerical rating scale (0–10) of stiffness	1	ALL
Pain and stiffness locations	Pain shaded in hips and shoulders to define PMR.	Manikin (Figure 2)	2	ALL
Daily stiffness pattern	Periods of day with stiffness in the past week	None/Morning/Lunchtime/ Afternoon/Early evening/ Late evening/During the night	1	ALL
Morning stiffness	Duration of stiffness from time of waking in the past week	None/1 to 15 minutes/16 to 45 minutes/ 46 minutes to an hour/More than an hour	1	ALL
Delay in patient consultation	Symptom chronicity at presentation to GP	Less than a week/1 to 2 weeks/ 2 to 4 weeks/More than 4 weeks	1	BL
Delay in GP diagnosis	Number of consultations with GP before receiving diagnosis	1/2/3/4 or more	1	BL
Shoulder movement	Ability to raise both arms above head at time of questionnaire	Yes/No	1	ALL
Patient perceived recovery	Patient’s self-perceived rating of recovery	6 category scale (Completely recovered through to Much worse)	1	1FU, 4FU, 8FU, 12FU, 18FU, 24FU
**PMR Medications**				
Cortico steroid dose	Current daily dose of prednisolone	Free text	1	ALL
Other medication use	Reported use of (i) Paracetamol, (ii) paracetamol + codeine, (iii) non-steroidal anti-inflammatories, (iv) strong analgesics, (v) gastro-intestinal protection, (vi) calcium + vitamin D, (vii) osteoporosis treatment, (viii) alternative therapies, (ix) anti-depressants, (x) other	Yes/No (free text for Other)	10	BL
**General health and other medical problems**				
General health	Self-rated health at time of questionnaire Global health rating	EuroQoL [16] Excellent/Very good/ Good/Fair/Poor	5 1	BL, 12FU, 24FU ALL
Falls	Falls in previous 12 months	Yes/No	1	BL, 12FU, 24FU
Osteoporosis	Ever fractured (i) hip, (ii) wrist, (iii) spine/vertebrae, (iv) other	Yes/No	4	BL, 12FU, 24FU
PMR exclusion symptoms	Reports of recent (i) Sudden headache, (ii) Tender scalp, (iii) Disturbed/double vision, (iv) Jaw claudication, (v) Temperature, (vi) Appetite loss, (vii) Unintentional weight loss, (viii) Joint swelling, (ix) Other	Yes/No (free text for Other)	9	BL
Dyspepsia	Bothersomeness of dyspepsia symptoms (ulcer, wind, indigestion, heartburn) at time of questionnaire Seeking of healthcare of dyspepsia symptoms (if present)	Not at all bothered/Bothered a little/ Bothered a lot Did not seek help/ Went to GP/Went to hospital/ Other type of care	1 1	4FU, 12FU, 24FU 4FU, 12FU, 24FU
**Daily activities**				
Activities of daily living	Performance in daily activities in past week	Modified Health Assessment Questionnaire [17] adapted to UK English [18]	8	ALL
**Fatigue and sleep**				
Fatigue	Fatigue symptoms in past week	Functional Assessment of Chronic Illness Therapy-Fatigue (Version 4) [19]	13	BL, 1FU, 12FU, 24FU
Sleep	Insomnia symptoms in past 2 weeks (including Diagnostic and Statistical Manual of Mental Disorders insomnia definition)	Insomnia Severity Index [20]	7	BL, 1FU, 12FU, 24FU
**Anxiety and depression**				
Generalised anxiety disorder	Anxiety symptoms in past two weeks	Generalized Anxiety Disorder-7 [21]	7	BL, 1FU, 12FU, 24FU
Depression	Depression symptoms in past two weeks	PHQ-8 [22]	8	BL, 1FU, 12FU, 24FU
**Socio-demographics**				
Age	Age at diagnosis	Date of birth	1	ALL
Sex	Sex	Male/Female	1	ALL
Ethnicity	Ethnicity	White/Mixed, multiple ethnic groups/ Asian, Asian British/Black, African, Caribbean, Black British/Other	1	BL
Employment status	Employment status at time of questionnaire	Employed/ Unemployed, seeking work/ House-wife/Retired/Not working due to ill health/Other	1	BL, 1FU, 4FU, 12FU, 18FU, 24FU
Work situation	Level of work at time of questionnaire (in those in reporting being employed as their employment status)	Doing usual job/ Working fewer hours/ Paid sick leave/Paid annual leave, holiday/ Doing lighter duties/Unpaid leave	1	BL, 10FU, 4FU, 12FU, 18FU, 24FU
Socioeconomic status	Occupational class based on (i) current or (ii) most recent job title	Job title – categorised as manual/ non-manual according to SOC 2010 [23]	1	BL
**Lifestyle**				
Smoking	Smoking status at time of questionnaire	Never/Previous/Current	1	BL
Alcohol	Frequency of drinking at time of questionnaire	Daily or almost daily/ 3 or 4 times a week/ Once or twice a week/ 1 to 3 times a month/ Special occasions only/Never	1	BL
Obesity	BMI at time of questionnaire	Height (m/ft) Weight (kg/st, lb)	1 1	BL BL, 12FU, 24FU
**Relationships and social support**				
Living arrangement	Live alone	Yes/No	1	BL, 24FU
Marital status	Marital status at time of questionnaire	Married/Separated/Divorced/Widowed/ Cohabiting/Single	1	BL, 12FU, 24FU
PMR interference with sexual relationships	Interference with intimate and sexual relationships	Not applicable/Not at all/ Little bit/ Moderately/Quite a bit/Extremely (adapted from [24])	1	BL, 12FU, 24FU
Social support	Availability of instrumental support Availability of emotional support	Yes/No/No need [25] Yes/No/No need	1 1	BL, 12FU, 24FU
**Views on PMR and information on the condition**				
Origin PMR	Cause of PMR	Free text	1	BL
Information regarding PMR	Information given by GP GP information from GP useful Desire for more information from GP Searched elsewhere for information	Yes/No Yes/No Yes/No Yes/No	1 1 1 1	BL
Worries regarding PMR	Flares	Yes/No	1	24FU
	Medications	Yes/No	1	
	Reducing medications	Yes/No	1	
	Side-effects of medication	Yes/No	1	
		Yes/No	1	
	Long-term consequences of PMR	Yes/No	1	
	Other			
Potentially useful for PMR patients in the future	Information	Yes/No and Received Yes/No	2	24FU
	Medications	Yes/No and Received Yes/No	2	
	Alternative medications	Yes/No and Received Yes/No	2	
		Yes/No and Received Yes/No	2	
	Complementary therapies	Yes/No and Received Yes/No Yes/No and Received Yes/No	2	
	Physiotherapy	Yes/No and Received Yes/No	2	
	Referral to secondary care		2	
	Other			

ALL = All follow-ups; BL = Baseline; 1FU = 1-month follow-up; 4FU = 4-month follow-up; 8FU = 8-month follow-up; 12FU = 12-month follow-up; 18FU = 18-month follow-up; 24FU = 24-month follow-up

**Figure 2:**
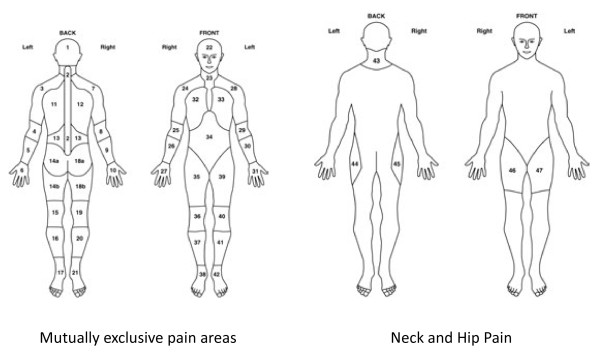
**Pain and stiffness manikins: definition of hips and shoulders.** Shoulders are defined as areas 3, 7, 24 and 28. Hips are defined as areas 44, 45, 46 and 47 [26].

#### Data entry, coding, cleaning and storage

Data will be pseudonymised and entered into a database specifically designed for this study. Prior to data entry, this database will be tested using a set of dummy data. Data will be entered as the completed questionnaires are received by dedicated members of the administration team. They are experienced in data entry. The Principal Investigator will determine coding prior to data entry. The database will provide coding options, to facilitate the entry of data. Some standard codes (e.g. missing data (−9), not applicable (−88)) are used by the Research Centre and will be utilised in this study.

A different member of the team will then check every tenth questionnaire as part of the quality assessment process. This information is kept by the Research Support Co-ordinator. Data will be stored in a password protected database and overseen by the Study Data Custodian. Only members of the research team will have access to the data. Requests for access to the data must be made in writing, along with an analysis plan, to the Principal Investigator. Completed questionnaires will be securely stored separately from the fax and consent forms, to protect the confidentiality of participants.

#### Sample size

PMR is a relatively rare condition in primary care and so the size of this study is constrained by practicalities to ensure a representative sample of patients for whom good quality data can be collected. From the CiPCA database [27], it can be estimated that for every 10,000 primary care patients aged 40 years and over, approximately 11 will receive a diagnosis of PMR in a 12-month period (Kelvin Jordan – personal communication). Using a stricter definition to define PMR, similar to that to be applied in this study, Smeeth et al [3] estimated that 8 per 10,000 patients in this age group experience a new onset of PMR every year. With an average practice list size of 5,000 (approximately half of whom will be aged 40 years or over), a two-year recruitment period, in 200 practices should enable identification of 1,100 patients with a Read-coded diagnosis of PMR, of whom 800 would be expected to have definite PMR according to the Smeeth definition [3]. Based on previous experience of this type of study in our Research Centre [28,29], we expect that 75 % of patients will respond to the questionnaire with 80 % of these consenting to have their medical records reviewed. This will result in an initial sample of 600 patients, with 480 forming the cohort that has agreed to medical record review. Allowing for loss to follow-up of 5 % per annum, this should leave a sample of approximately 430 patients at the end of the two year follow-up period.

#### Statistical analysis

A descriptive summary of those consenting and not consenting to join the cohort will be provided, as will a description of loss-to-follow-up in the cohort over the two years of the study.

The psychometric properties of the Modified Health Assessment Questionnaire (MHAQ) [17] will be investigated to assess its suitability for use in this population. The recently proposed clinical criteria for remission and relapse in PMR [11] will also be implemented to investigate their feasibility in primary care epidemiological studies.

With these findings in mind, regression models will be used to describe the natural history and prognosis of PMR over a two-year period. These models will take into account PMR-related disability, as well as quality of life, sexual functioning and work disability, and will incorporate both self-reported constructs and information derived from medical records. The average daily dose of corticosteroids will be calculated and modelled as a potential prognostic factor for disease activity and new onset comorbidities.

An overall prognostic model for the chosen outcome in PMR over two years will be derived and tested.

Where necessary, multiple imputation techniques will be used to assess the impact of loss-to-follow-up.

## Discussion

To our knowledge, there has been no previous primary care inception cohort study of PMR. This study will fill this gap, by collecting detailed information via postal questionnaires and medical record review (where consent is given), from patients receiving a new primary care diagnosis of PMR. This study will provide guidance on the optimal methods of outcome assessment for PMR in primary care, and will develop new prognostic models, relevant to clinical practice in this group. This study should ultimately lead to improved outcomes for older patients diagnosed with this common inflammatory condition.

## Competing interests

The authors declare that they have no competing interests.

## Authors’ contributions

CDM and SH conceived the idea for the study. All authors participated in the design of the study. SM drafted the manuscript, which was approved by all authors.

## Pre-publication history

The pre-publication history for this paper can be accessed here:

http://www.biomedcentral.com/1471-2474/13/102/prepub
